# Symptom Clustering Patterns and Population Characteristics of COVID-19 Based on Text Clustering Method

**DOI:** 10.3389/fpubh.2022.795734

**Published:** 2022-02-04

**Authors:** Xiuwei Cheng, Hongli Wan, Heng Yuan, Lijun Zhou, Chongkun Xiao, Suling Mao, Zhirui Li, Fengmiao Hu, Chuan Yang, Wenhui Zhu, Jiushun Zhou, Tao Zhang

**Affiliations:** ^1^Sichuan Center for Disease Control and Prevention, Chengdu, China; ^2^Department of Epidemiology and Health Statistics, West China School of Public Health and West China Fourth Hospital, Sichuan University, Chengdu, China; ^3^Anyue County Center for Disease Control and Prevention, Ziyang, China

**Keywords:** COVID-19, symptom clustering patterns, risk factor, time delay, epidemiology

## Abstract

**Background:**

Descriptions of single clinical symptoms of coronavirus disease 2019 (COVID-19) have been widely reported. However, evidence of symptoms associations was still limited. We sought to explore the potential symptom clustering patterns and high-frequency symptom combinations of COVID-19 to enhance the understanding of people of this disease.

**Methods:**

In this retrospective cohort study, a total of 1,067 COVID-19 cases were enrolled. Symptom clustering patterns were first explored by a text clustering method. Then, a multinomial logistic regression was applied to reveal the population characteristics of different symptom groups. In addition, time intervals between symptoms onset and the first visit were analyzed to consider the effect of time interval extension on the progression of symptoms.

**Results:**

Based on text clustering, the symptoms were summarized into four groups. *Group 1: no-obvious symptoms*; *Group 2: mainly fever and/or dry cough*; *Group 3: mainly upper respiratory tract infection symptoms*; *Group 4: mainly cardiopulmonary, systemic, and/or gastrointestinal symptoms*. Apart from Group 1 with no obvious symptoms, the most frequent symptom combinations were fever only (64 cases, 47.8%), followed by dry cough only (42 cases, 31.3%) in Group 2; expectoration only (21 cases, 19.8%), followed by expectoration complicated with fever (10 cases, 9.4%) in Group 3; fatigue complicated with fever (12 cases, 4.2%), followed by headache complicated with fever was also high (11 cases, 3.8%) in Group 4. People aged 45–64 years were more likely to have symptoms of Group 4 than those aged 65 years or older (odds ratio [*OR*] = 2.66, 95% *CI*: 1.21–5.85) and at the same time had longer time intervals.

**Conclusions:**

Symptoms of COVID-19 could be divided into four clustering groups with different symptom combinations. The Group 4 symptoms (i.e., mainly cardiopulmonary, systemic, and/or gastrointestinal symptoms) happened more frequently in COVID-19 than in influenza. This distinction could help deepen the understanding of this disease. The middle-aged people have a longer time interval for medical visit and was a group that deserve more attention, from the perspective of medical delays.

## Introduction

The coronavirus disease 2019 (COVID-19) has evolved into a global pandemic, causing significant morbidity and mortality worldwide. As of December 2021, it has caused more than 270 million confirmed cases and more than 5 million deaths worldwide, with the number of confirmed cases continues to increase at a rate of about 100,000 per day (1).

Clinical symptoms, as indicators for the identification and diagnosis, play a vital role in the early detection and treatment. COVID-19 has a wide range of clinical manifestations, ranging from asymptomatic to severe viral pneumonia (2, 3). It has been widely confirmed that fever, dry cough, expectoration, and fatigue were the most common symptoms in patients with COVID-19 (3–5). As the pandemic progressed, symptoms of cardiovascular system (6), digestive system (7), petechial skin rash (8), and loss of taste (ageusia) and smell (anosmia) (9) were also reported. Numerous studies have contributed to the understanding of COVID-19. Despite a growing body of evidence in this field, the heterogeneity in both individuals and studies still left much to explore about the symptomatology of COVID-19.

For the clinical symptoms, most previous works have been primarily descriptive studies and focused on descriptions of single symptoms (4, 5). Noting the variability of symptoms and there are normally two or more symptoms coexisted in one infected case, the association and aggregation of different symptoms may provide more information. The purpose of this study was to explore whether there were potential clustering patterns of different symptoms in patients with COVID-19 based on the aggregation of symptoms with a text clustering method. On the basis of clustering results, we examined the population characteristics of different symptom groups. Given that there were both overlaps and variations in symptoms of COVID-19 and other infectious diseases, such as influenza (10–13), we also compared the symptom groups found in this study with symptoms of influenza reported in other studies. By profiling the symptoms of COVID-19 and its population characteristics, we expect to provide some inspiration for enhancing the understanding of people of the disease's clinical manifestations and identifying the high frequent symptom combinations of COVID-19.

## Materials and Methods

### Study Design and Data Source

In this retrospective cohort study, a total of 1, 067 laboratory confirmed cases of COVID-19 from January 21, 2020 to November 20, 2020 in Sichuan Province were included. Demographic information, symptoms onset, comorbidities, and epidemiological data of all cases were extracted from individual epidemiological investigation report sourced from the Epidemic Registration System of the Sichuan Center for Disease Control and Prevention (CDC). The symptoms were first pre-recorded in the form of the epidemiological investigation report, and for self-reported symptoms not included in the form, they were appended as a free text by the CDC colleagues. Epidemiological data included dummy variables, such as whether a case was an indigenous case or an imported case from abroad, and the variable about whether a case had been infected individually or had been infected in a clustered family or workplace. This study was approved by the Ethics Committee of Sichuan Center for Disease Control and Prevention (SCCDCIRB-2020-007). Written informed consent was obtained from each of subjects.

### Statistical Analysis

First, with the symptoms text of cases, the k-means clustering method was used to explore the potential symptom groups on the basis of Euclidean distance. The optimal number of clusters was determined by the widely accepted elbow method (14). Bar charts were used to give a visual representation of the symptom combinations under each group. Categorical variables were represented by counts and percentages, continuous variables in nonnormal distribution were represented by median (interquartile ranges, IQR), otherwise by mean ± SD.

Based on the clustering results, with symptom groups as the dependent variable, a multinomial logistic regression was applied to identify potential factors associated with the symptom groups. Group 1 was the reference category in the multinomial regression model. Population characteristics, such as age, gender, comorbidities (hypertension, diabetes, lung disease, and cardiovascular disease), and epidemiological characteristics (imported or indigenous, clustered or individual) were added into the model as covariates. According to Tian et al. (15), the ages were cut into four groups: aged 0–12, 13–44, 45–64, and ≥65 years. Due to lack of comorbidities and epidemiological information, considering the small proportion of missing, we depicted some respondents in the demographic description, yet not included them in the regression model. Besides, time intervals between symptoms onset and the first visit were depicted also the proportions of different symptom groups at different time intervals were visualized by a bar diagram.

**Figure 1:**
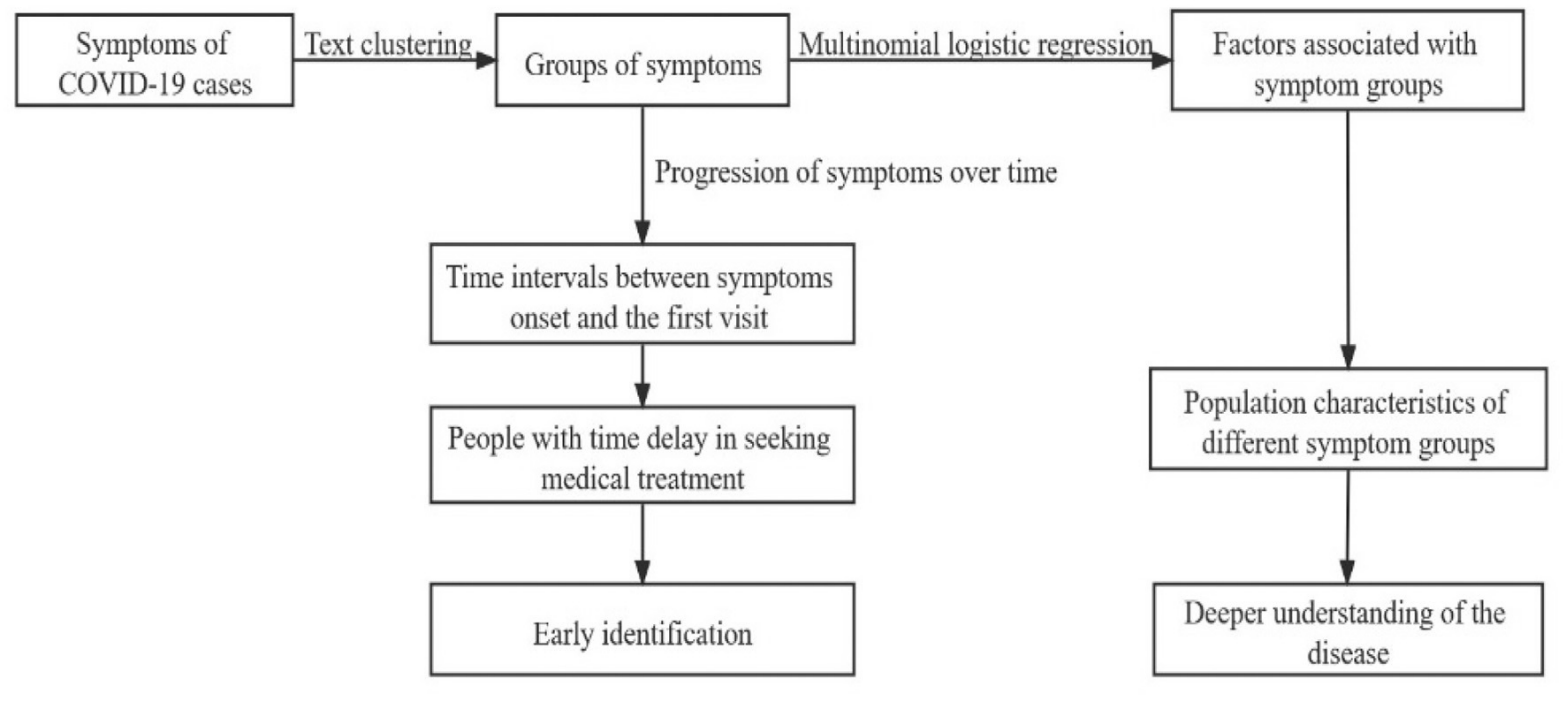
The procedure of analysis in this study.

Figure 1 shows the procedure of our analysis. In this study, the text clustering was conducted with Python version 3.7.6 and the rest statistical analyses were conducted with R version 4.0.3. The value of *p* < 0.05 was considered statistically significant.

## Results

### Population Distribution and Symptom Clustering Patterns

From January 21, 2020 to November 20, 2020, information of 1,067 cases was collected. The majority of infected cases were in 13–44 years (613 cases, 57.45%) and 45–64 years (344 cases, 32.23%) age groups. For comorbidities, the prevalence of hypertension was 6.84%, while it was 2.44, 3.00, and 2.36% of diabetes, lung disease, and cardiovascular disease, respectively. In addition, 41.24% of the infected patients were imported cases and 26.43% were infected with family clustering (Table 1).

**Table 1:** Characteristics of cases in different symptoms groups.

**Characteristics**	**Group 1**	**Group 2**	**Group 3**	**Group 4**	**Total**
	**(*n =* 541)**	**(*n =* 134)**	**(*n =* 106)**	**(*n =* 286)**	**(*n =* 1,067)**
Age group, *n* (%)					
0–12	26 (65.00)	5 (12.50)	6 (15.00)	3 (7.50)	40
13–44	349 (56.93)	69 (11.26)	50 (8.16)	145 (23.65)	613
45–64	140 (40.70)	47 (13.66)	41 (11.92)	116 (33.72)	344
≥65	26 (37.14)	13 (18.57)	10 (14.29)	21 (30.00)	70
Gender					
Male	388 (55.51)	84 (12.02)	61 (8.73)	166 (23.75)	699
Female	153 (41.58)	50 (13.59)	45 (12.23)	120 (32.61)	368
Hypertension					
Yes	23 (31.51)	9 (12.33)	10 (13.70)	31 (42.47)	73
No	511 (51.83)	125 (12.68)	95 (9.63)	255 (25.86)	986
Unidentified^*^	7 (87.50)	0 (0.00)	1 (12.50)	0 (0.00)	8
Diabetes					
Yes	2 (7.69)	3 (11.53)	5 (19.23)	16 (61.54)	26
No	532 (51.50)	131 (12.68)	101 (9.78)	269 (26.04)	1,033
Unidentified^*^	7 (87.50)	0 (0.00)	1 (12.50)	0 (0.00)	8
Lung disease					
Yes	7 (21.88)	8 (25.00)	2 (6.25)	15 (46.87)	32
No	527 (51.31)	126 (12.27)	104 (10.13)	270 (26.29)	1027
Unidentified^*^	7 (87.50)	0 (0.00)	1 (12.50)	0 (0.00)	8
Cardiovascular disease					
Yes	5 (20.00)	4 (16.00)	3 (12.00)	13 (52.00)	25
No	529 (51.16)	130 (12.57)	103 (9.96)	272 (26.31)	1,034
Unidentified^*^	7 (87.50)	0 (0.00)	1 (12.50)	0 (0.00)	8
Imported cases					
Yes	354 (80.45)	40 (9.09)	23 (5.23)	23 (5.23)	440
No	187 (29.82)	94 (14.99)	83 (13.24)	263 (41.95)	627
Clustered cases					
Yes	146 (51.77)	40 (14.18)	34 (12.06)	62 (21.99)	282
No	324 (48.80)	81 (12.20)	58 (8.73)	201 (30.27)	664
Unidentified^*^	71 (58.68)	13 (10.74)	14 (11.57)	23 (19.01)	121

* *Missing values*.

The elbow method indicated that the sum of squares within a group was minimal when the data were divided into four groups. Therefore, four clusters were selected for the analysis. Then, combined with pathophysiology (16, 17) and consultation from clinical experts in the Sichuan Center for Disease Control and Prevention, the symptoms were summarized as follows: *Group 1: no-obvious symptoms*, referred to those with no obvious symptoms but positive nucleic acid test; *Group 2: mainly fever and/or dry cough*, referred to those with fever as the main symptoms, or complicated with dry cough; *Group 3: mainly upper respiratory tract infection symptoms*, referred to those mainly with expectoration and upper respiratory tract infection symptoms, such as pharyngodynia, stuffy nose and runny nose, or complicated with fever; *Group 4: mainly cardiopulmonary, systemic, and/or gastrointestinal symptoms*, referred to those whose main symptoms were cardiopulmonary symptoms, such as shortness of breath, dyspnea, chest tightness, chest pain, and/or systemic symptoms, such as fatigue, chills, and myalgia, and/or symptoms of the gastrointestinal system, such as nausea, vomiting, and diarrhea, sometimes accompanied by fever and upper respiratory tract symptoms.

The results showed that more than half (50.7%) of the infected cases did not show obvious symptoms (Group 1) at the first visit. For the three groups with obvious symptoms, their proportions were 12.6%, 10.0%, and 26.8%, respectively. Among them, Group 4, i.e., cardiopulmonary, systemic, and/or gastrointestinal symptoms had higher proportion. Population characteristics of the above symptom groups are summarized in Table 1.

**Figure 2:**
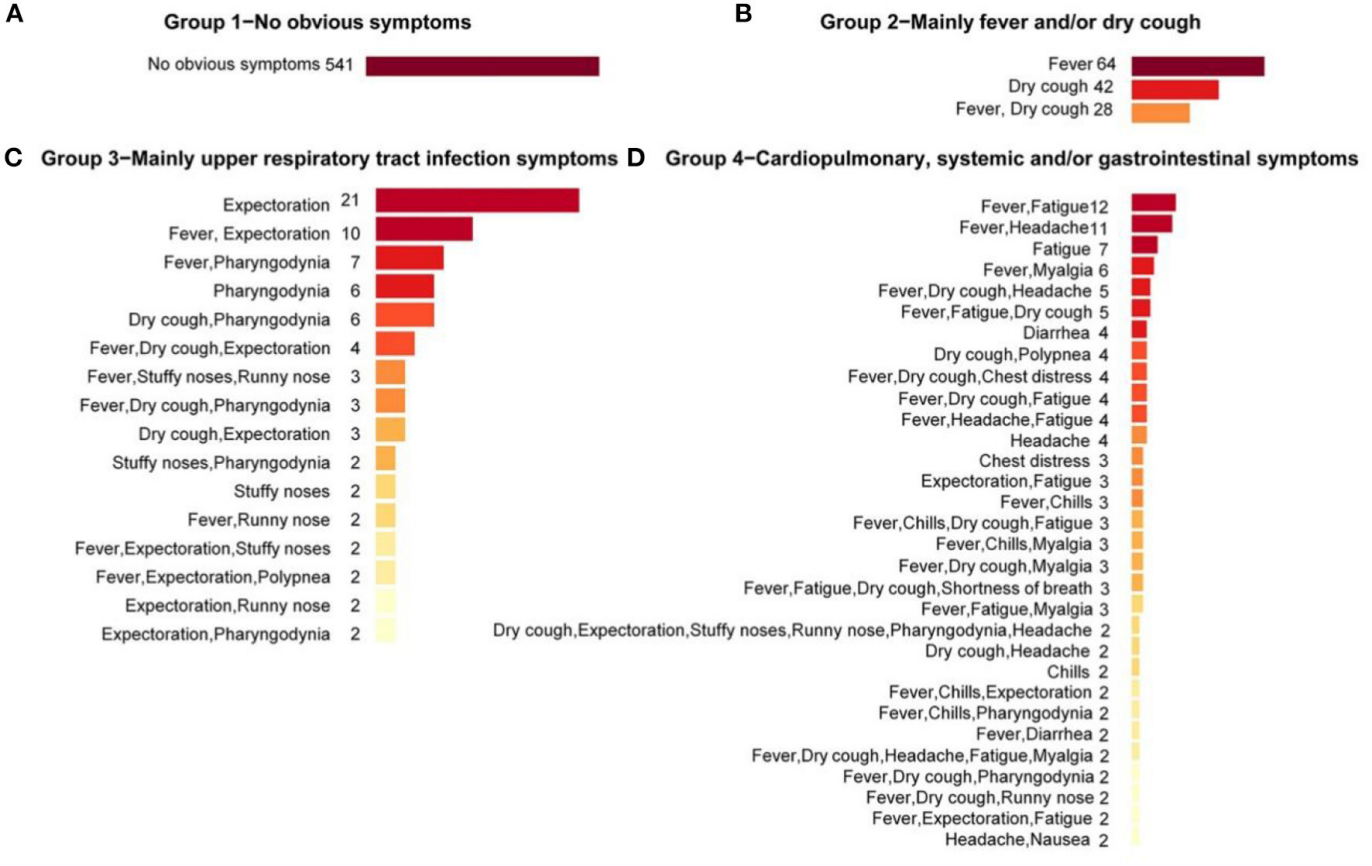
Symptom combinations under different symptom groups. Symptom combinations with only one case in symptom Groups 3 and 4 were not included. **(A)** The Group 1 with no obvious symptoms; **(B)** The Group 2 with main symptoms were fever and/or dry cough; **(C)** The Group 3 with main symptoms were upper respiratory tract infection symptoms; **(D)** The Group 4 with main symptoms were cardiopulmonary, systemic and/or gastrointestinal symptoms.

To profile the symptoms composition under each group, bar charts were applied to visualize the particular symptom combinations under each group (Figure 2). It could be seen that there were overlaps and interactions of symptoms under a same group. In symptom Group 1, all cases were with no-obvious symptoms (541 cases, 100%). In symptom Group 2, the most frequent symptom combinations were fever only (64 cases, 47.8%), followed by dry cough only (42 cases, 31.3%). In symptom Group 3, the most frequent symptom combinations were expectoration only (21 cases, 19.8%), followed by expectoration complicated with fever (10 cases, 9.4%). In symptom Group 4, the most frequent symptom combinations were fatigue complicated with fever (12 cases, 4.2%), the incidence of headache complicated with fever was also high (11 cases, 3.8%). In general, except for the asymptomatic with the highest proportion (50.70%), the six most frequent symptom combinations in the whole population were fever only (6.00%), dry cough only (3.94%), dry cough complicated with fever (2.62%), expectoration only (1.97%), fatigue complicated with fever (1.12%), and headache complicated with fever (1.03%).

As for the dominant single symptom, in general, fever and dry cough were the two most frequent symptoms, with frequencies of 64.4% and 38.8%, respectively, followed by expectoration (12.0%) and fatigue (11.4%). Under the groups, fever (68.7%) and dry cough (52.24%) were the dominant symptoms in Group 2; Expectoration (59.4%) and pharyngodynia (29.24%) were the dominant symptoms in Group 3; and fatigue (42.7%) and headache (26.2%) were the dominant symptoms in Group 4. Under the groups, symptoms showed some clustering around the dominant symptoms.

### Population Characteristics of Different Symptom Groups

The results of univariate and multivariate multinomial logistic regression assessing the population characteristics of different symptom groups are shown in Table 2. In the univariable analysis, higher age, female, and comorbidities (hypertension, diabetes, lung ailment, and cardiovascular disease) were all associated with increased odds of the presence of symptoms of Group 4, namely symptoms, such as cardiopulmonary, systemic, and/or gastrointestinal symptoms. The imported cases and cases infected with family clustering had lower odds of symptoms in all the three groups of obvious symptoms.

**Table 2:** Results of population characteristics of different symptom groups.

	**OR (95% CI)**		**OR (95% CI)**		**OR (95% CI)**	
	**Univariate**	**Multivariate**	**Univariate**	**Multivariate**	**Univariate**	**Multivariate**
Age (years)						
13–44 vs. 0–12	1.03 (0.38–2.8)	1.21 (0.42–3.45)	0.59 (0.23–1.51)	0.74 (0.27–2.03)	3.73 (1.11–12.55)	4.08 (1.13–14.76)
45–64 vs. 0–12	1.97 (0.71–5.43)	1.89 (0.65–5.49)	1.35 (0.52–3.52)	1.29 (0.46–3.61)	7.94 (2.34–26.98)	5.91 (1.61–21.7)
≥65 vs. 0–12	2.49 (0.75–8.23)	1.49 (0.41–5.44)	1.7 (0.52–5.49)	0.84 (0.23–3.1)	7.54 (1.98–28.7)	2.22 (0.51–9.7)
Gender						
Female vs. male	1.47 (0.97–2.22)	0.91 (0.58–1.44)	1.67 (1.06–2.63)	0.96 (0.58–1.59)	1.65 (1.20–2.25)	0.82 (0.56–1.21)
Comorbidities						
Hypertension yes vs. no	1.51 (0.65–3.51)	0.87 (0.34–2.23)	2.01 (0.86–4.69)	1.04 (0.39–2.75)	2.66 (1.48–4.77)	1.27 (0.59–2.73)
Diabetes yes vs. no	7.88 (0.71–87.68)	7.69 (0.63–94.66)	26.65 (3.08–230.89)	29.43 (3.00–288.66)	30.51 (4.02–231.42)	41.72 (4.56–381.92)
Lung disease yes vs. no	4.06 (1.40–11.81)	2.04 (0.66–6.34)	1.45 (0.30–7.11)	0.58 (0.11–3.09)	4.02 (1.62–9.98)	1.49 (0.53–4.23)
Cardiovascular disease yes vs. no	1.96 (0.35–10.82)	1.18 (0.2–7.15)	3.88 (0.85–17.65)	2.28 (0.45–11.65)	6.08 (1.96–18.85)	3.64 (0.97–13.73)
Imported cases						
Yes vs. no	0.23 (0.15–0.35)	0.2 (0.12–0.33)	0.15 (0.09–0.26)	0.13 (0.07–0.24)	0.05 (0.03–0.09)	0.04 (0.02–0.06)
Clustered cases						
Yes vs. no	1.1 (0.72–1.68)	0.59 (0.36–0.95)	1.34 (0.84–2.13)	0.58 (0.34–0.98)	0.67 (0.48–0.95)	0.27 (0.17–0.41)

Additionally, the multivariate regression model showed that compared with the 0–12 years age group, the odds of symptoms of Group 4 increased in both the 13–44 years and 45–64 years age groups (odds ratio [*OR*] = 4.08, 95% *CI*: 1.13–14.76; *OR* = 5.91, 95% *CI*: 1.61–21.7). Furthermore, if the group of ≥65 years was changed as the reference group, it could be derived from the results of multinomial logistic regression that people aged 45–64 years were more likely to develop symptoms of Group 4 (*OR* = 2.66, 95% *CI*: 1.21–5.85) when compared with the ≥65 years group. The plausibility of this result would be discussed in the discussion section. No significant differences in the odds of showing symptoms of the three obvious groups were detected between male and female. For the comorbidities, the odds of showing symptoms of Group 2 had no significant differences between patients with and without diabetes (*OR* = 7.69, 95% *CI*: 0.63–94.66), but in those with diabetes, the odds of showing symptoms of Group 3 and Group 4 significantly escalated (*OR* = 29.43, 95% *CI*: 3.00–288.66; *OR* = 41.72, 95% *CI*: 4.56–381.52), indicating diabetes as a strong risk factor for upper respiratory tract symptoms, cardiopulmonary, systemic, and/or gastrointestinal symptoms. In addition, the results showed that there was no significant difference in the odds of all the three obvious symptom groups between patients with or without hypertension (*OR* = 1.27, 95% *CI*: 0.59–2.73), lung disease (*OR* =1.49, 95% *CI*: 0.53–4.23), or cardiovascular disease (*OR* = 3.64, 95% *CI:* 0.97–13.73). Besides, the results showed that, the incidences of all the three obvious symptom groups were lower in the imported cases and the patients infected with family clustering than in the indigenous cases and non-clustering cases, respectively (*OR* < 1, *p* < 0.05).

### Time Intervals Between Symptoms Onset and the First Visit

**Figure 3:**
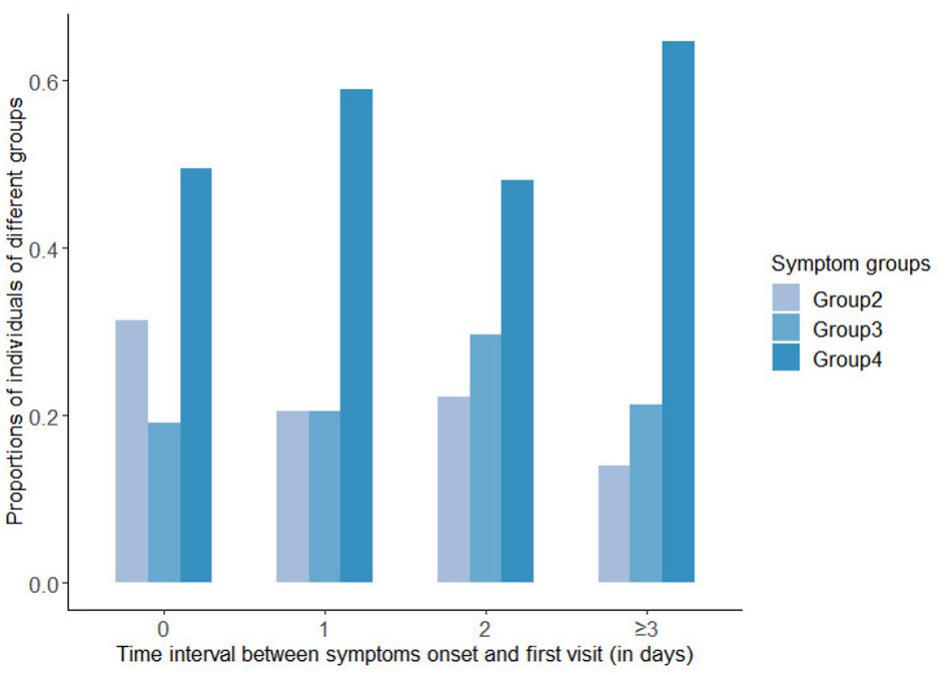
Bar chart of proportions of the groups with obvious symptoms at different time intervals.

In all the symptomatic cases, the median time interval between symptoms onset and the first visit was 1 day, and the IQR was (0,3) days. In addition, 47.5% of symptomatic patients visited a medical institution on the day of symptoms onset, 15.4% visited 1 day after onset, 11.4% visited 2 days after onset, and 25.7% sought medical treatment 3 days or more after onset. Figure 3 displayed the proportions of the three groups with obvious symptoms at different time intervals. It could be seen that the proportion of symptoms of Group 2 was decreasing as the time interval lengthened, while in Group 4, it was increasing over longer time intervals, and in Group 3, the proportion peaked at the intermediate time.

**Figure 4:**
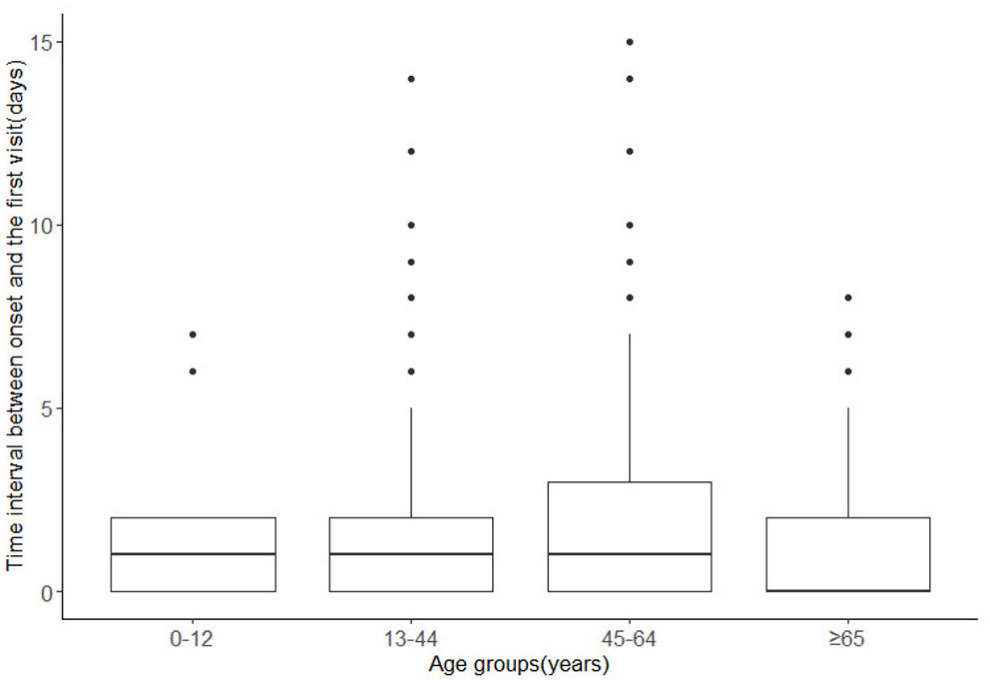
Time intervals between symptoms onset and the first visit in different age groups.

The analysis of time intervals in different age groups showed that the median time intervals in 0–12, 13–44, and 45–64 years old groups were all 1 day, while it was 0 day in ≥65 years age group (Figure 4). The ranges were larger in 13–44 years age group and 45–64 years age group, with ranges of (0,14) days and (0,15) days respectively, while the ranges in 0–12 years and ≥65 years age group were (0,7) days and (0,8) days, respectively. Patients aged 13–64 years seemed to have longer time intervals.

## Discussion

This study focused on the aggregation of different symptoms of COVID-19, and explored the potential symptoms clustering patterns. Similar to many previous studies (2–5, 18), we found that fever and dry cough were the most common symptoms, followed by expectoration and fatigue. Besides that, this study found there existed probable clustering patterns of symptoms, which could be summarized into four groups. Furthermore, the common symptom combinations under each group were illustrated. Specifically, the most frequent symptom combinations under the three groups with obvious symptoms (Group 2, Group 3, and Group 4) were fever only, expectoration only, and fatigue accompanied with fever, respectively.

It has been confirmed that both COVID-19 and influenza have fever, cough, and expectoration as their main symptoms (13, 19, 20). However, distinction between the two was that symptoms, such as vomiting, stuffy nose, runny nose, and ocular symptoms were more common in influenza than in COVID-19 (10, 11, 21). In COVID-19, symptoms such as fatigue, neurological symptoms (headache), gastrointestinal symptoms (diarrhea), and acute respiratory distress syndrome (ARDS) (chest distress) occurred more frequently (22–24). Similar conclusions were reached in a systematic review comparing COVID-19 and influenza (12). These distinct symptoms were largely consistent with those clustered into Group 4 in this study (i.e., mainly cardiopulmonary, systemic, and/or gastrointestinal symptoms), under which the four most frequent symptom combinations were fatigue complicated with fever, headache complicated with fever, fatigue only, and myalgia complicated with fever. Given there were both overlaps and variations between COVID-19 and influenza, information from single symptoms was limited. Therefore, awareness of the symptoms clustering patterns and the commonly accompanying symptoms may provide more information for enhancing the understanding of this disease.

Besides, the population characteristics in different symptom groups assessed with multinomial logistic regression showed that compared with the younger age groups (0–12 years), those aged 13–44, 45–64, and ≥65 years had increased odds of showing symptoms of Group 4. This has been confirmed in previous studies that immunosenescence and inflamm-aging may be an explanation (25, 26). For the comorbidities, patients with chronic diseases, such as diabetes were more likely to show symptoms of Group 4, which has been confirmed (27). In addition, the results showed that for the imported cases and the clustered cases, the odds of symptoms of Group 2, Group 3, and Group 4 were all lower than indigenous cases or non-clustered cases, respectively. For the imported cases, the entry quarantine for the imported (28) may provide an explanation. Additionally, for the results that cases infected with clustering were less likely to show more severe symptoms, this may be reasonable that infection occurred within a same family, work unit, nursery, or school means an infected person was more likely to be found as a close contact of whom with which the person was clustered, and thus was more likely to be found at the early stage and showed milder symptoms at the first clinical visit.

For the result that the prevalence of symptom Group 4 (26.8%) was higher than that of Group 2 (12.6%) and Group 3 (10.0%), this study took consideration of the progression of symptoms over time. From the results of the time intervals analysis, the proportion of symptom Group 2 decreased as the time interval extended, while the proportion of Group 4 increased. This indicated that the presence of symptom Group 4, to some extent, may be related to a longer time interval between symptoms onset and the time infected individuals sought medical treatment. Infected individuals who sought medical treatment later were more likely to had symptoms of Group 4. These results were partly supported by several previous studies focusing on the dynamics of symptoms. According to Larsen et al. (29), a study on the symptoms in 55,924 confirmed cases based on a Markov process showed that there was a possible order in the development of COVID-19 symptoms. The symptoms may progress initially with fever or cough followed by upper respiratory symptoms, such as sore throat, after fatigue and other systemic symptoms, and gastrointestinal symptoms, such as nausea, vomiting, diarrhea, and abdominal pain were presented at a later stage of the disease. Huang et al. (30) analyzed the clinical characteristics of 305 patients in the early stage of the pandemic in Wuhan Jinyintan Hospital, China. They found that compared with symptoms in the early stages of disease, as the time interval lengthened, the incidence of cardiopulmonary symptoms increased significantly. A similar pattern was found in the work of Mizrahi et al. (31). These results reflected that longer interval may indicate a higher possibility of gastrointestinal symptoms (such as, nausea, vomiting, and diarrhea), cardiopulmonary symptoms (such as, shortness of breath and dyspnea), and/or systemic symptoms, which were largely consistent with the symptoms of Group 4 in this study.

Another concern was that the odds of symptom Group 4 was higher in patients aged 45–64 years than in aged ≥65 years. Despite the immunosenescence and inflamm-aging (32), elderly people were not as likely to show more severe initially symptoms as expected. However, the influence of symptoms progression may not be neglected. Results in this study showed that people aged 45–64 years have more cases with longer time intervals, indicating a time delay for medical treatment in this population. Similarly, a study of 14,168 hospitalized infected cases in Belgium found that working age group (aged 20–60 years) had longer intervals between symptoms onset and their visit to a doctor than the elderly people in nursing homes (33). One plausible explanation was that for the elderly people, any abnormal body signal may be more likely to be detected than the working population because they usually pay more attention to their health than the latter. In contrast, the middle-aged people were more likely to have longer time delay for medical visit than the elderly people, and as a result, had more severe symptoms when first diagnosed. Thus, considering the time-delay effect, this study suggested that middle-aged people, may be a subpopulation deserving special attention in the prevention and control of the epidemic. Measures, such as health dissemination can be taken to improve the timeliness of medical treatment for the working-age population. Besides, the employers could also relieve the work-related stresses through the provision of paid time-off.

In contrast to many studies that mainly described only single symptoms, this study focused on the associations among different symptoms, and explored the potential symptoms clustering patterns. Besides, it was found that the presences of different groups of symptoms may be related to the time intervals between symptoms onset and the time infected individuals sought medical treatment. These results provided us a further understanding of the spectrum of COVID-19 symptoms. Furthermore, this study revealed that people of working age were more likely to have a time delay for medical treatment, as a result, had higher possibility of showing symptoms of Group 4. This could provide inspiration for targeted prevention and control of COVID-19.

This study had several limitations. First, for comorbidities, information, such as severity and duration, was not collected, so the impact of comorbidities may be biased by the heterogeneity of severity grade and duration of the diseases. In addition, in the analysis of the population characteristics of different symptom groups, taking diabetes as an example, the *OR* value and its *CI* were large, which was attributed to the small number of cases answering “Yes.” For these results, though statistically significant, the conclusions were still imprecise and unclear, so more research is needed in the future. Second, for the self-reported symptoms, there may be memory bias. As individuals may have deep memories of some symptoms or ignore others. With the spread of the pandemic, in the late pandemic, such as in November or summer, individuals may delay the consultation or neglect and consider more of influenza rather than COVID-19. Similarly, there may be information bias of the self-reported time of symptom onset. Therefore, more efforts in the future will be needed to validate these findings and turn them into COVID-19 combating practice. Furthermore, it should also be noted that all the patients in this study were infected before the end of November 2020. Therefore, for some variants of severe acute respiratory syndrome coronavirus 2 (SARS-CoV-2) discovered afterward, such as Gamma (34), Delta (35), Omicron (36) and possible future variants, the results of this study would not be directly applicable. However, it is expected that our analysis procedure might be taken as reference in the future as further variants arise.

## Conclusions

This study focused on the associations of symptoms of COVID-19 and found that the symptoms could be divided into four different clustering groups. The Group 4 symptoms clustered in this study, that were mainly cardiopulmonary, systemic, and/or gastrointestinal symptoms, happened more frequently in COVID-19 than in influenza. This distinction could help deepen the understanding of this disease. In addition, we found that the middle-aged population may be a group requiring more attention during this epidemic, and some measures, such as paid time-off are expected to improve the timeliness of medical treatment for this group.

## Data Availability Statement

The raw data supporting the conclusions of this article will be made available by the authors, without undue reservation.

## Ethics Statement

The studies involving human participants were reviewed and approved by the Ethics Committee of Sichuan Center for Disease Control and Prevention (SCCDCIRB-2020-007). Written informed consent to participate in this study was provided by the participants' legal guardian/next of kin.

## Author Contributions

XC, HW, and TZ conceptualized the analysis. XC and HW implemented statistical analysis. XC, HW, HY, JZ, and TZ contributed to the study implementation, interpretation of results, and writing of the manuscript. LZ, CX, SM, ZL, FH, CY, and WZ did the data collection and cleaning. All authors reviewed and provided comments on the manuscript and approved the final version.

## Funding

This work was supported by the National Natural Science Foundation of China (82041033, 81602935), the Sichuan Science and Technology Program (2020YFS0015, 2020YFS0091, and 2021YFS0001), the Health Commission of Sichuan Province (20PJ092), the Chongqing Science and Technology Program (cstc2020jscx-cylhX0003), the Chengdu Science and Technology Program (2021-YF05-01585-SN), the Sichuan University (2018hhf-26), the Central Government Funding Items (Grant Numbers 2021zc02), and Liangshan Yi Autonomous Prefecture Center for Disease Control and Prevention (H210322). The funding body did not participate in the design, collection, analysis, interpretation, and writing of this study.

## Conflict of Interest

The authors declare that the research was conducted in the absence of any commercial or financial relationships that could be construed as a potential conflict of interest.

## Publisher's Note

All claims expressed in this article are solely those of the authors and do not necessarily represent those of their affiliated organizations, or those of the publisher, the editors and the reviewers. Any product that may be evaluated in this article, or claim that may be made by its manufacturer, is not guaranteed or endorsed by the publisher.
